# Which women stop smoking during pregnancy and the effect on breastfeeding duration

**DOI:** 10.1186/1471-2458-6-195

**Published:** 2006-07-26

**Authors:** Roslyn C Giglia, Colin W Binns, Helman S Alfonso

**Affiliations:** 1 Curtin University of Technology, School of Public Health, GPO Box U1987, Perth WA 6845, Australia

## Abstract

**Background:**

Cigarette smoking during pregnancy increases the risk of adverse pregnancy outcomes and women who quit smoking at this time are able to reduce the risk of low birth weight, preterm labour, spontaneous abortion and perinatal death. This study investigates the socio-demographic characteristics of pregnant women who stop smoking during pregnancy and the association between stopping smoking and breastfeeding duration.

**Methods:**

A 12 month longitudinal study was conducted in two public maternity hospitals in Perth, Australia between mid-September 2002 and mid-July 2003. While in hospital, participating mothers completed a self-administered baseline questionnaire. Follow up telephone interviews were conducted at 4, 10, 16, 22, 32, 40 and 52 weeks.

**Results:**

A total of 587 (55%) mothers participated in the study. Two hundred and twenty six (39%) mothers reported smoking prior to pregnancy and 77 (34%) of these stopped smoking during pregnancy. Women who were pregnant for the first time were twice as likely (OR = 2.05; 95% CI 1.047 – 4.03; p < 0.05) to quit smoking as multiparous women. Women who smoked more than 10 cigarettes per day were significantly less likely to quit smoking during pregnancy (OR = 0.36; 95% CI 0.18 – 0.69; p < 0.05). Women who consumed alcohol before pregnancy were three times more likely to quit smoking (OR = 2.58; 95% CI 1.00 – 6.66; p < 0.05). Quitting smoking during pregnancy was significantly associated with breastfeeding for longer than six months (OR = 3.70; 95% CI 1.55 – 8.83; p < 0.05).

**Conclusion:**

Pregnancy is a time when many women are motivated to quit smoking and providing targeted smoking cessation interventions at this time, which take into account factors predictive of quitting smoking, are more likely to be successful.

## Background

Despite considerable public understanding of the dangers of smoking during pregnancy, prevalence levels in Australia range between approximately 17%, reported in 2001, and 35%, reported in 1996 [1,2].

Substantial public health gains remain to be made in perinatal mortality and morbidity through the reduction of smoking during pregnancy [3] and pregnancy appears to be a time when women are highly motivated to quit smoking in the best interests of their unborn foetus. However, despite this not all women choose to quit smoking at this time and the differences between women who do stop smoking during pregnancy and those who don't may be caused by factors that can be influenced.

Lu et al reviewed nine cohort studies and found that the determinants of smoking cessation during pregnancy included maternal age, parity, number of cigarettes per day and duration of smoking, education level, partner's smoking status and socioeconomic status [4]. Furthermore, Ershoff, Solomon and Dolan-Mullen found additional sociodemographic and psychosocial differences between women with low intentions to stop smoking and those with high intentions [5]. In order to develop successful maternal smoking cessation public health programs the major determinants of quitting smoking during pregnancy need to be incorporated into intervention efforts.

The research team has already reported a significant increase over a 10 year period in the number of women breastfeeding upon discharge from hospital in Perth, Western Australia [6]. Likewise the major determinants of breastfeeding duration have been identified from the Perth Infant Feeding Study (PIFSII) cohort study [7]. The aims of this study were to document the number of women stopping smoking during pregnancy and to further examine the factors influencing the ability to stop smoking at this time. In addition, consideration was given to the exploration of variables (alcohol use before and during pregnancy; and attendance at antenatal classes) not previously reported in the Australian literature. The relationship between stopping smoking during pregnancy and breastfeeding duration was also examined.

## Methods

The second Perth Infant Feeding Study (PIFSII) was conducted between mid-September 2002 and mid-July 2003 to monitor breastfeeding rates and identify changes in breastfeeding practices and the determinants of breastfeeding. The study was conducted in the same hospitals using the same methodology as the first Perth Infant Feeding Study (PIFSI). PIFSI was conducted 10 years previous and results have been reported elsewhere [8].

Mothers were contacted within the first three days following the birth of their infant. Women were considered eligible for the study if they had delivered a live infant free of any serious health conditions requiring transfer to the neonatal intensive care unit at Perth's major maternity hospital. Mothers whose infants were admitted to the Special Care Nurseries (SCN) of the participating hospitals were eligible for recruitment.

Those women agreeing to participate in the study completed the self-administered baseline questionnaire while in hospital or shortly after discharge. Women declining to participate were asked to provide some basic sociodemographic data in order to determine the representativeness of the sample. All women regardless of their chosen infant feeding method were followed up by telephone interview at 4, 10, 16, 22, 32, 40 and 52 weeks postpartum. The study instruments used were essentially the same as that used in PIFSI, with only minor improvements and additions being made to the instruments used in the PIFSII. Questions relating to smoking were based on the 1989–90 National Health Survey [9]. Mothers were asked if they had smoked before pregnancy and if they had smoked during pregnancy as part of the baseline questionnaire. Mothers who acknowledged that they had smoked before pregnancy but had not smoked during pregnancy were categorised as 'stopping smoking' during pregnancy.

### Statistical analysis

Data were entered and analysed using the Statistical Package for Social Sciences, Version 11.0 (SPSS for Windows, SPSS Inc., Chicago, IL, USA). Risk factors associated with stopping smoking during pregnancy were analysed using the baseline questionnaire. Variables identified in the literature as being associated with breastfeeding initiation and duration were examined and included in the development of each statistical model.

Estimation of odds ratios was performed for univariate analysis testing statistical significance by χ^2 ^test. Adjusted odds ratios were calculated by logistic regression. All variables presented in Table 2 were entered into the model for the multivariate analysis of predicting stopping smoking during pregnancy. The model was reduced manually by excluding those variables with a less significant value.

The difference between duration of breastfeeding in those who stopped smoking during pregnancy and those who did not was initially explored using Kaplan Meier survival analysis. This relationship was further examined using logistic regression to examine breastfeeding duration less than and greater than six months using a variety of sociodemographic, biomedical and psychosocial factors reported to have an effect on breastfeeding duration in the literature. Variables were entered into the model to determine the effect on breastfeeding duration for more than or less than six months. Non-significant variables (p > 0.10) were manually excluded from the final model. Further analysis of this relationship using a Cox proportional hazard model was not attempted as the proportionality assumption had been violated and additional potential analytical techniques were considered beyond the scope of this paper. The six month time period was chosen based on the WHO recommendations for exclusive breastfeeding and was considered to be a significant reference point for infant feeding duration.

A mother's attitude towards infant feeding was measured by the Iowa Infant Feeding Attitude Scale (IIFAS) [10]. The IIFAS is a 17 item scale which measures attitudes towards both breast and formula feeding with regards to the health and nutritional benefits, and the cost and convenience of each method. It has been shown previously to be a valid and reliable measure of infant feeding attitudes amongst women in the USA [10] and Scotland [11]. Each item is measured on a 5-point scale and total scores could range from 17 (reflecting positive formula feeding attitudes) to a high of 85 (indicating attitudes that favour breastfeeding). For the purposes of the analysis mothers were split into two groups, those with an IIFAS score at or above the median (≥ 65) and those with a score less than the median (<65).

Presented *p *values are two-sided, and a 5% significance level was used.

### Ethical considerations

The PIFSII was approved by the Human Ethics Committee of Curtin University and the Research Ethics Committees of the two participating hospitals. Signed informed consent was obtained from participants. Confidentiality was assured and mothers were advised that their participation was voluntary and that they could withdraw at any time without prejudice.

## Results

In the PIFSII, 870 women of the 1068 women eligible to participate were contacted and 587 completed baseline questionnaires (55%) and were maintained throughout the study period, representing 68% of women contacted. Those women discharged from hospital within the first 24 hours or not on the ward at the times that the researcher visited account for the eligible women not contacted. A similar proportion of eligible women participated in the PIFSI (58%) and the PIFSII (55%) studies. No significant differences were found in the age or level of education of participants compared with non-participants in either study [6,12].

A total of 226 women were smoking before pregnancy. This represents 39% of the total population of mothers. The proportion of mothers smoking decreased to 25% (n = 149) during pregnancy. Of the women who reported smoking before they became pregnant (n = 226), 34% of these (n = 77) stopped smoking during pregnancy, a reduction of 14%. Table 1 outlines the prevalence of smoking mothers and their partners.

Table 2 shows the results of the univariate analysis of smoking cessation during pregnancy using variables previously reported to be important and other relevant demographic variables. Stopping smoking during pregnancy was significantly associated with primiparous women (OR = 2.6; 95% CI 1.5–4.5; p < 0.05). A woman was less likely to stop smoking during pregnancy if she had a partner who smoked, had less than 12 years of education, smoked more than ten cigarettes per day, had an unplanned pregnancy and did not attend antenatal classes. Age, income level and mother's country of origin were not significantly associated with stopping smoking during pregnancy. Drinking alcohol before pregnancy was significantly associated with stopping smoking during pregnancy. However drinking alcohol during pregnancy was not associated with stopping smoking during pregnancy.

Table 3 shows the results of the multivariate analysis used to determine which variables were independent predictors of stopping smoking during pregnancy. It indicates that women who were primigravida and women who drank alcohol before pregnancy were more likely to stop smoking during pregnancy. Women who smoked more than ten cigarettes per day were less likely to stop smoking during pregnancy.

The association between stopping smoking and breastfeeding duration was explored using multivariate analysis. Table 4 indicates that stopping smoking during pregnancy was significantly related to breastfeeding duration. Of the 77 women who stopped smoking during pregnancy 35 (45%) of these continued to breastfeed for longer than six months. One hundred and forty nine women continued to smoke during pregnancy and of these, 34 (23%) breastfed for longer than six months. Women who stopped smoking were almost four times more likely to breastfeed for longer than six months, after adjustment for potential confounders (OR = 2.8; 95% CI 1.6 – 5.1; p < 0.05, adjusted OR = 3.7; 95% CI 1.6 – 8.8; p < 0.05).

## Discussion

In this study approximately 34% of women who smoked before pregnancy reported stopping smoking during pregnancy. This is slightly higher than figures reported in the 1999 – 2002/3 National Tobacco Strategy of 20 to 30%, [13] however since this time smoking cessation rates in pregnancy may have increased in line with the general community [14]. In the analysis of the Australian Longitudinal Study on Women's Health year 2000 dataset a figure of 55% of women quitting was reported [15]. Most recently however a figure of 4% of women quitting smoking during pregnancy has been reported by Moshin and Bauman [16] in a large cross sectional study in New South Wales, Australia. Internationally figures range from 15.8% of women quitting smoking during pregnancy from a national survey in Canada [17] to 26.8% from New Zealand [18]. The disparity in these prevalence levels is most likely due to the timing of the data collection and whether the method of survey is cohort based or cross-sectional [19].

The relationship between smoking cessation during pregnancy and breastfeeding duration for longer than six months postpartum has not previously been reported in the research literature. More commonly continued maternal smoking in pregnancy has been reported in association with reduced breastfeeding initiation and duration [20-22], and only one previous study has explored smoking status and breastfeeding duration up to 26 weeks [23]. In this study women who stopped smoking during pregnancy were significantly more likely to breastfeed for longer than six months, which is in accordance with national and international recommendations [24,25]. Although stopping smoking is not exclusively responsible for prolonged breastfeeding duration, [26,27] promoting smoking cessation during pregnancy supports both positive perinatal outcomes and supports optimal breastfeeding duration, known to be associated with protection against infection, some chronic diseases and improved cognitive development in the infant [25,28].

The reported effects of alcohol consumption as a predictor of smoking cessation during pregnancy are varied and inconsistent, however in this study consuming alcohol prior to pregnancy was significantly associated with stopping smoking during pregnancy. In previous research Severson et al [19] looked at alcohol in the week prior to the study questionnaire being administered (administered two weeks postpartum) and found that mothers who stopped smoking during pregnancy were less likely to have consumed alcohol in this week. Using data from the US National Maternal and Infant Health Survey, consuming one or more drinks during pregnancy was independently associated with a lower likelihood of quitting smoking during pregnancy [29] and in a study of the relationship between quitting tobacco, alcohol and caffeine consumption during pregnancy, Pirie et al found that quitting either alcohol or cigarettes was not associated with an increased likelihood of quitting the other substance (e.g. quitting alcohol was not associated with quitting smoking or vice versa). Although this relationship was not significant in the multivariate model the clustering of multiple substance use in individuals was [30]. More recently a subset population of women in Spain who consumed alcohol (time of consumption not confined to either during pregnancy or three months after the birth) were also found to have a lower chance of quitting smoking [31].

In contrast, early research conducted in Sweden found continued alcohol consumption during pregnancy was not associated with a decrease in mothers stopping smoking [32]. Similarly, data from Canada demonstrated that drinking during pregnancy was positively related to a woman's likelihood of attempting to quit smoking during pregnancy [17]. However alcohol consumption was also significantly associated with cessation relapse before the child was born and the authors propose that although more women who drink make cessation attempts they are also more likely to relapse as it may be too difficult to give up smoking and drinking alcohol at the same time, or that continued alcohol use impairs the cessation maintenance. More recently, a New Zealand study found that women who quit smoking in the first trimester were more likely to report alcohol consumption at this time compared to women who reported not consuming alcohol [18].

In the current study alcohol intake before and during pregnancy was considered with regard to smoking cessation during pregnancy. The associations between alcohol before and during pregnancy with stopping smoking during pregnancy have previously not been studied concurrently. We found that women who consumed alcohol before pregnancy were more likely (OR = 2.6;95% CI 1.0–6.7; p < 0.049) to stop smoking during pregnancy. Alcohol intake is a health risk behaviour known to cluster with cigarette smoking [30,33] and therefore it is likely that those women who are consuming alcohol are also smoking hence the women most likely to stop smoking are those women drinking alcohol. Alcohol consumption during pregnancy was not significantly related to smoking cessation at this time.

In accordance with previous research, a woman was more likely to stop smoking during pregnancy if she was primigravida [16,18,29-31,34-38]. Women who have smoked during a previous pregnancy generally have an experience of giving birth to one or more healthy children and are therefore less motivated to quit smoking for subsequent pregnancies.

Pre-pregnancy smoking levels indicate that women who quit smoking during pregnancy are probably less addicted to smoking than women who continue to smoke. In the present study a woman was more likely to stop smoking if she reported smoking less than 10 cigarettes per day in the pre-pregnancy period. This result conforms with the current literature in that women who smoke at low levels are more likely to quit smoking [19,30,35-37,39,40].

Having a partner who smokes [19,31,35,36,38] and a low level of education [19] are factors previously found to be predictive of continued smoking during pregnancy. Although significant at the univariate level, education and father's smoking status were no longer significant when included in the multivariate analysis. Interestingly, a greater number of fathers stopped smoking after the baby was born. This may be due to the perception that the baby does not seem 'real' until after the birth when fathers are prompted by the baby's presence to quit smoking [41].

Antenatal classes aim to prepare expectant parents for childbirth and their new family life. Attendance at antenatal classes was a significant predictor of smoking cessation in the univariate analysis, although not significant in the multivariate model. Previous studies have found that early attendance at antenatal care was predictive of stopping smoking during pregnancy [16,35].

Timing of the pregnancy as a predictive factor for stopping smoking during pregnancy has not previously been reported using Australian data. Internationally previous research has shown that women having an unplanned pregnancy were more likely to continue smoking during pregnancy [32], whereas others have failed to find an effect of an unintended pregnancy [29]. In this study, women whose pregnancy was unplanned were less likely to stop smoking during pregnancy, however this was not significant in the multivariate analysis. A planned pregnancy enables a woman the opportunity to consider stopping smoking in preparation for the antenatal period, whereas women who become pregnant unexpectantly have less time to implement this change.

Neither age nor income was related to the likelihood of stopping smoking during pregnancy. The correlation with age has been found in some previous studies [19] but not in others [38-40]. A relationship between age and smoking cessation during pregnancy is still unclear and further research is required in this area.

Studies have reported 66% higher medical costs attributed to complicated births for smoking mothers compared with non-smoking mothers [42]. In Australia it has been estimated that smoking during pregnancy is responsible for 78 infant deaths, 6890 hospital separations and a cost of AUD23 million dollars to the health care system each year [43].

As smoking cessation programs have been shown to reduce the odds of continued smoking in pregnancy [44], it is imperative that the factors found in this study and previous research to predict smoking cessation during pregnancy be addressed in evidence based intervention programs. This concurs with recommendations from the 2001 National Tobacco Strategy [45]. However despite this recommendation there appears to be a lack in the provision of any routine antenatal smoking cessation advice in the Australian health care setting [46,47].

This study did not define those women who quit in the pre-pregnancy period from those who quit during pregnancy, often referred to as 'spontaneous quitters' [48]. In addition, there is considerable evidence outlining a high prevalence of relapse in the postpartum period in women who quit smoking during pregnancy, [39] however the design of this research study did not enable this issue to be addressed. Future cohort studies should take smoking abstinence into consideration in the design phase. Consideration of factors contributing to residual confounding, such as emotional antenatal attachment to the foetus in relation to smoking cessation, were not measured in this research. Future research should examine these additional potential factors that may help further explain the relationship between pregnancy and smoking cessation.

As in most studies of smoking during pregnancy, all smoking behaviours were self-reported in this study and cigarette smoking may have been underreported particularly during the antenatal period when there is an increased stigma associated with smoking. Nevertheless, self-reported smoking status is considered to be reasonably accurate [49,50] and results presented here give a good picture of smoking during pregnancy. Although this study used a standardised questionnaire, to elicit smoking information, future studies should consider the inclusion of alternative measures of cigarette smoking.

A further limitation of the study is having less than 60% of eligible women participate. Nevertheless, the sample size is still relatively large (>500), and there was no significant difference in maternal age and level of education between participant and non-participants, suggesting that the sample was representative of the population from which it was drawn.

Notwithstanding the relatively small sample size, and the fact that all women came from government-based hospitals, results from this study do reflect the current evidence. In addition these mothers are representative of the 'hard to reach' groups in Australian health promotion. Therefore the lessons learned from this study could be usefully applied in health education programs.

## Conclusion

Quitting smoking during pregnancy is a potential area for huge public health gain in the short term through decreasing smoking related harm, and in the long term by promoting the positive health benefits of prolonged breastfeeding. Pregnancy is a time when women are more receptive to quitting smoking and many opportunities exist for implementing cessation efforts that are succinct and simple. A large proportion of women stop smoking voluntarily at this time, however many continue putting their health and that of their unborn foetus at risk. The current study highlights women who are primiparous, smoke less than 10 cigarettes per day before pregnancy, and consume alcohol before pregnancy, as significant predictors of quitting smoking at this time. Quitting smoking during pregnancy is supportive of breastfeeding for longer than six months.

In an effort to decrease the risk of adverse pregnancy outcomes and promote the best possible health outcomes for the infant and the mother, smoking cessation intervention programs in pregnancy should be designed with the predictive factors identified in this study in mind. It is also important that tobacco control strategies targeting the mainstream population run concurrently with smoking cessation programs for pregnant women. Antenatal care services at all levels and in both the public and private domain are paramount in supporting these cessation efforts.

## Authors' contributions

RCG had primary responsibility for the data analysis and writing the manuscript.

CWB supervised the design and execution of the study, and contributed to writing the manuscript.

HA participated in the final data analyses and contributed to writing the manuscript.

## Competing interests

The author(s) declare that they have no competing interests.

## Pre-publication history

The pre-publication history for this paper can be accessed here:



## Figures and Tables

**Table 1:** Smoking history of women (n = 226) and their partners (n = 270) (%)

**Smoking History**	**Before Pregnancy**		**During Pregnancy**		**Week 4 postpartum**	
	
	**Women**	**Partners**	**Women**	**Partners**	**Women**	**Partners**
Smoked	226 (100)	270 (100)	149 (66)	245 (91)	122 (54)	172 (64)
Stopped smoking	N/A	N/A	77 (34)	25 (4)	17 (8)	N/A

N/A – This data not available.

**Table 2:** Characteristics of women who did and did not stop smoking in pregnancy (n = 226). Figures are percentages if not otherwise stated

	Women who stopped smoking (n = 77)	Women who did not stop smoking (n = 149)	*p value*	Univariate OR (95% CI)
<25 years	46.8	35.6	0.103	1.6 (0.9–2.8)
>25 years	53.2	64.4		1
Income <$25 000	56	65.8	0.156	0.7 (0.4–1.2)
Income >$25 000	44	34.2		1
Primiparous	55.8	32.9	**0.001**	**2.6 (1.5–4.5)**^†^
Multiparous	44.2	67.1		1
Caucasian	90.9	96.6	0.068	0.3 (0.1–1.1)
Non-caucasian	9.1	3.4		1
Father smoked in pregnancy	61	75.5	**0.025**	**0.5 (0.3–0.9)**^†^
Father did not smoke in pregnancy	39	24.5		1
<12 years education	46.1	62.8	**0.016**	**0.5 (0.3–0.9)**^†^
>12 years education	53.9	37.2		1
Drank alcohol before pregnancy	87	72.3	**0.012**	**2.6 (1.2–5.5)**^†^
Non-drinker before pregnancy	13	27.7		1
Alcohol during preg	40.3	38.9	0.846	1.1 (0.6–1.9)
No alcohol in preg	59.7	61.1		1
Cigs before preg <10/day	63.6	36.4		1
Cigs before preg >10/day	36.4	63.6	**<0.001**	**0.3 (0.2–0.6)**^†^
Timing of pregnancy			**0.045**	
Planned	44	37.3		1
Mistimed	41.3	32.4		1.1 (0.6–2.0)
Unplanned	14.7	30.3		**0.4 (0.2–0.9)**^†^
Attend antenatal classes	33.8	52		1
Did not attend antenatal classes	66.2	48	**0.009**	**0.5 (0.3–0.8)**^†^

^†^Significant at *p *= 0.05. Significant figures in bold

**Table 3:** Multivariate analysis of factors predicting likelihood of stopping smoking during pregnancy (n = 77)

	**OR of stopping smoking during pregnancy (CI 95%)**	***p***
Before pregnancy >10 cigarettes/day	0.4 (0.2–0.7)	0.002
Primiparous	2.1 (1.0–4.0)	0.036
Drank alcohol before pregnancy	2.6 (1.0–6.7)	0.049

Variables in the full model included age, income, mother's nationality, whether father smoked during the pregnancy, maternal years of education and whether the mother attended antenatal classes.

**Table 4:** Stopping smoking during pregnancy and breastfeeding for longer than 6 months (n = 77)

Variable	OR breastfeeding >6 months (95% CI)	*p*
Stopping smoking in pregnancy^a^	2.8 (1.6–5.1)	0.001
Stopping smoking in pregnancy^b, c^	3.7 (1.6–8.8)	0.003

^a^unadjusted

^b^reference group is women who did not stop smoking

^c^adjusted for income, age of infant when mother returned to work, breastfeeding problems at or before week four postpartum, age of infant when pacifier first used and mothers infant feeding attitude (IIFAS).

## References

[B1] Centre for Epidemiology and Research New South Wales Department of Health (2002). Mothers and babies, 2001. NSW Public Health Bulletin.

[B2] Australian Longitudinal Study on Women's Health. http://www.newcastle.edu.au/centre/wha/.

[B3] US Department of Health and Human Services (2001). Women and smoking: a report of the Surgeon General – 2001. Office on Smoking and Health, National Center for Chronic Disease Prevention and Health Promotion, Centers for Disease Control and Prevention Washington, DC: Office on Smoking and Health, National Center for Chronic Disease Prevention and Health Promotion, Centers for Disease Control and Prevention.

[B4] Lu Y, Tong S, Oldenburg B (2001). Determinants of smoking cessation during and after pregnancy. Health Promot Int.

[B5] Ershoff DH, Solomon LJ, Dolan-Mullen P (2000). Predictors of intentions to stop smoking early in prenatal care. Tob Control.

[B6] Graham KI, Scott JA, Binns CW, Oddy WH (2005). National targets for breastfeeding at hospital discharge have been achieved in Perth. Acta Paediatr.

[B7] Scott JA, Binns CW, Oddy WH, Graham KI (2006). Predictors of breastfeeding duration: evidence from a cohort study. Pedatrics.

[B8] Scott JA, Aitkin I, Binns CW, Aroni RA (1999). Factors associated with the duration of breastfeeding amongst women in Perth, Australia. Acta Paediatr.

[B9] Australian Bureau of Statistics (1991). National Health Survey questionnaire 1989–1990. Canberra;.

[B10] De la Mora A, Russell D, Dungy C, Losch M, Dusdieker L (1999). The Iowa Infant Feeding Attitude Scale: analysis of reliability and validity. Journal of Applied Social Psychology.

[B11] Scott JA, Shaker I, Reid M (2004). Parental attidtudes toward breastfeeding: Their association with feeding outcome at hospital discharge. Birth.

[B12] Scott JA, Binns CW, Aroni RA (1997). The influence of reported paternal attitudes on the decision to breast-feed. J Paediatr Child Health.

[B13] McDermott L, Russell A, Dobson A (2002). Cigarette smoking among women in Australia. National Tobacco Strategy 1999–2002/03 occasional paper.

[B14] Australian Institute of Health and Welfare (2005). 2004 National Drug Strategy Household Survey: first results. AIHW cat. no. PHE 57. Canberra: AIHW.

[B15] McDermott L, Dobson A, Russell A (2004). Changes in smoking behaviour among young women over life stage transitions. Aust N Z J Public Health.

[B16] Moshin M, Bauman AE (2005). Socio-demographic factors associated with smoking and smoking cessation among 426,344 pregnant women in New South Wales, Australia. BMC Public Health.

[B17] Connor SK, McIntyre L (1999). The sociodemographic predictors of smoking cessation among pregnant women in Canada. Can J Public Health.

[B18] McLeod D, Pullon S, Cookson T (2003). Factors that influence changes in smoking behaviour during pregnancy. N Z Med J.

[B19] Severson HH, Andrews JA, Lichtenstein E, Wall M, Zoref L (1995). Predictors of smoking during and after pregnancy: a survey of mothers of newborns. Prev Med.

[B20] Horta BL, Kramer MS, Platt RW (2001). Maternal smoking and the risk of early weaning: a meta-analysis. Am J Public Health.

[B21] Ludvigsson JF, Ludvigsson J (2005). Socio-economic determinants, maternal smoking and coffee consumption, and exclusive breastfeeding in 10205 children. Acta Paediatr.

[B22] Amir LH, Donath SM (2002). Does maternal smoking have a negative physiological effect on breastfeeding? The epidemiological evidence. Birth.

[B23] Liu J, Rosenberg KD, Sandoval AP (2006). Breastfeeding duration and perinatal cigarette smoking in a population-based cohort. Am J Public Health.

[B24] World Health Organization (2001). The optimal duration of exclusive breastfeeding. Report of an expert consultation. Geneva: WHO.

[B25] Binns CW, Davidson GP, NHMRC (2003). Infant feeding guidelines for health workers. Food for Health: Dietary Guidelines for Children and Adolescents in Australia.

[B26] Gilchrist D, Woods B, Binns CW, Scott JA, Gracey M, Smith H (2004). Aboriginal mothers, breastfeeding and smoking. Aust N Z J Public Health.

[B27] Leung GM, Lam T, Ho L (2002). Breast-feeding and its relation to smoking and mode of delivery. Obstet Gynecol.

[B28] Labbok MH, Clark D, Goldman AS (2004). Breastfeeding: maintaining an irreplaceable immunological resource. Nat Rev Immunol.

[B29] Kahn RS, Certain L, Whitaker RC (2002). A reexamination of smoking before, during, and after pregnancy. Am J Public Health.

[B30] Pirie PL, Lando H, Curry SJ, McBride CM, Grothaus LC (2000). Tobacco, alcohol, and caffeine use and cessation in early pregnancy. Am J Prev Med.

[B31] Torrent M, Sunyer J, Cullinan P, Basagana X, Harris J, Garcia O, Anto JM (2004). Smoking cessation and associated factors during pregnancy. Gac Sanit.

[B32] Dejin-Karlsson E, Hanson BS, Ostergren P, Ranstam J, Isacsson S, Sjoberg N (1996). Psychosocial resources and persistent smoking in early pregnancy – a population study of women in their first pregnancy in Sweden. J Epidemiol Community Health.

[B33] English RM, Najman JM, Bennett SA (1997). Dietary intake of Australian smokers and nonsmokers. Aust N Z J Public Health.

[B34] Cnattingius S, Lindmark G, Meirik O (1992). Who continues to smoke while pregnant?. J Epidemiol Community Health.

[B35] Panjari M, Bell RJ, Astbury J, Bishop SM, Dalais F, Rice GE (1997). Women who spontaneously quit smoking in early pregnancy. Australian and New Zealand Journal of Obstetrics and Gynaecology.

[B36] Hakansson A, Lendahls L, Petersson C (1999). Which women stop smoking?. Acta Obstetricia et Gynaecologica Scandinavica.

[B37] Lindqvist R, Aberg H (2001). Who stops smoking during pregnancy?. Acta Obstetricia et Gynaecologica Scandinavica.

[B38] Suzuki J, Kikuma H, Kawaminami K, Shima M (2005). Predictors of smoking cessation during pregnancy among the women of Yamato and Ayase municipalities in Japan. Public Health.

[B39] Wakefield MA, Jones WR (1991). Cognitive and social influences on smoking behaviour during pregnancy. Australian and New Zealand Journal of Obstetrics and Gynaecology.

[B40] Fingerhut LA, Kleinman JC, Kendrick JS (1990). Smoking before, during and after pregnancy. Am J Public Health.

[B41] Wakefield M, Reid Y, Roberts L, Mullins R, Gillies P (1998). Smoking and smoking cessation among men whose partners are pregnant: a qualitative study. Soc Sci Med.

[B42] Orleans CT, Barker DC, Kaufman NJ, Marx JF (2000). Helping pregnancy smokers quit: meeting the challenge in the next decade. Tob Control.

[B43] Ford JH, Dobson AJ (2004). Smoking and pregnancy. Med J Aust.

[B44] Lumley J, Oliver SS, Chamberlain C, Oakley L (2005). Interventions for promoting smoking cessation during pregnancy (review). Cochrane Database of Systematic reviews, Published in the Cochrane Library.

[B45] Miller M, Wood L (2001). Smoking cessation interventions. Review of evidence and implications for best practice in health care settings. National Tobacco Strategy 1999–2002/03 occasional paper.

[B46] Mabbutt J, Bauman A, Moshin M (2002). Tobacco use of pregnant women and their male partners who attend antenatal classes: what happened to routine quit smoking advice in pregnancy?. Aust N Z J Public Health.

[B47] Hunt JM, Lumley J (2002). Are recommendations about routine antenatal care in Australia consistent and evidence-based?. Med J Aust.

[B48] DiClemente CC, Dolan-Mullen P, Windsor RA (2000). The process ofpregnancy cessation: implications for interventions. Tob Control.

[B49] Klebanoff MA, Levine RJ, Morris CD, Hauth JC, Sibai BM, Curet LB, Catalano P, Wilkins DG (2001). Accuracy of self-reported cigarette smoking among pregnant women in the 1990s. Paediatric and Perinatal Epidemiology.

[B50] Heath AC, Knopik VS, Madden PA, Neuman RJ, Lynskey MJ, Slutske WS, Jacob T, Martin NG (2003). Accuracy of mother's retrospective reports of smoking during pregnancy: comparison with twin sister informant ratings. Twin Research.

